# Supplementary material for: skandiver: a divergence-based analysis tool for identifying intercellular mobile genetic elements
Source: Bioinformatics. 2024 Sep 4;40(Suppl 2):ii155–64. doi: 10.1093/bioinformatics/btae398 (PMC11373320; doi:10.1093/bioinformatics/btae398)
Supplement: btae398_Supplementary_Data [file btae398_supplementary_data.zip › skandiver-supplement-2024-05-26.pdf]

Supplemental Figures for skandiver: a divergence-based analysis tool for identifying intercellular mobile genetic elements

Xiaolei Brian Zhang, Grace Oualline, Jim Shaw, Yun William Yu

May 26, 2024

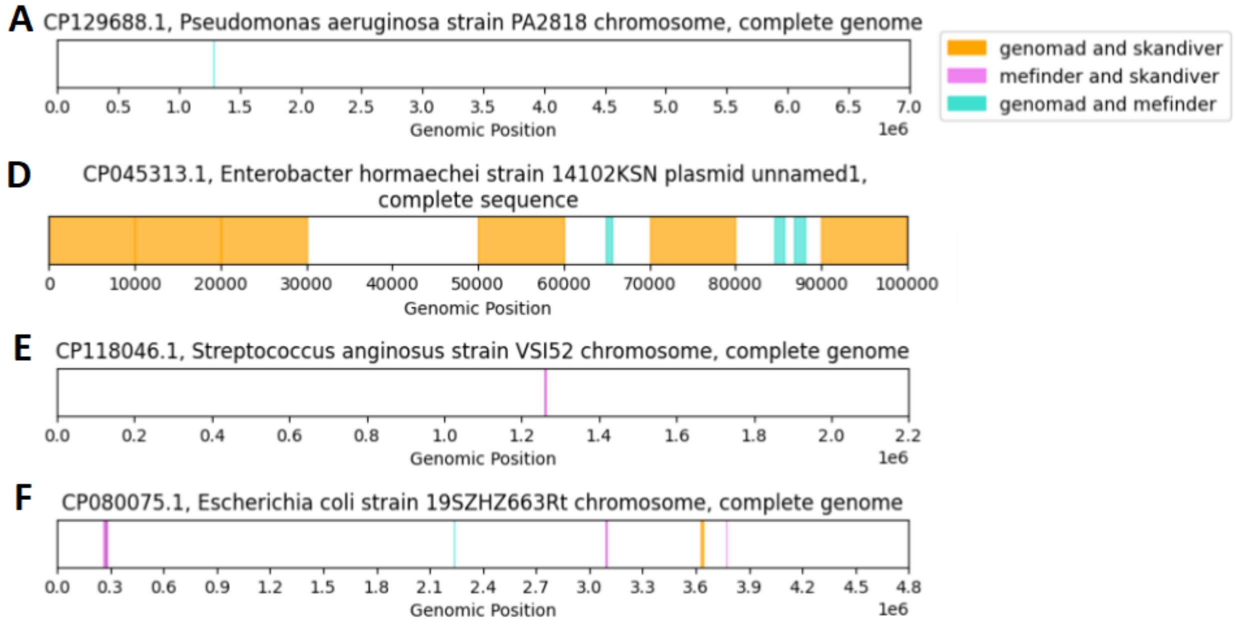

Supp Fig. 1: **Overlap of mobile element finding profiles of skandiver, MobileElementFinder (MEFinder), and geNomad: the three tools find different sets of putative genetic mobile elements.** Building off of Figure 3, we labeled areas where skandiver, MobileElementFinder, and geNomad found overlapping regions corresponding to potential mobile elements. Three of the seven genome assemblies shown in Figure 3 did not contain any regions of overlap between the three methods, and are not shown here; **A.** *Pseudomonas aeruginosa* strain PA2818 chromosome, **D.** *Enterobacter hormaechei* strain 14102KSN plasmid, **E.** *Streptococcus anginosus* strain VS152 chromosome, **F.** *Escherichia coli* strain 19SZHZ663Rt chromosome.
