# Supplementary material for: skandiver: a divergence-based analysis tool for identifying intercellular mobile genetic elements
Source: Bioinformatics. 2024 Sep 4;40(Suppl 2):ii155–64. doi: 10.1093/bioinformatics/btae398 (PMC11373320; doi:10.1093/bioinformatics/btae398)
Supplement: btae398_Supplementary_Data [file skandiver-ECCB-response-2024-05-26.pdf › skandiver-ECCB-response-2024-05-26.pdf]

2024-May-26

Dear Editor,

Thank you for giving us the opportunity to resubmit a revised version of our manuscript, "skandiver: a divergence-based analysis tool for identifying intercellular mobile genetic elements", which incorporates the reviewers' valuable suggestions to improve our manuscript.

We have addressed both reviewer reports in the point-by-point response below and incorporated their suggestions into our manuscript. We have also summarized the most important changes in the response to the metareview:

We have color-coded our response as follows:

Response legend:

Black - Reviewer comment
Green - Authors

We would like to express our appreciation and careful reading by the editors and referees of the ECCB conference. We believe their comments have improved our manuscript greatly. We hope that our work is now acceptable for ECCB and publication in Bioinformatics.

Sincerely,

Xiaolei Brian Zhang,
Grace Oualline,
Jim Shaw, and
Yun William Yu

Reviews

------------------------ METAREVIEW ------------------------

Notes: • *The one significant drawback of this study is the lack of a robust truth set to test the three tools against. Could such a dataset be constructed? It likely would be a challenge to construct, but without one, conclusions about sensitivity or accuracy must be moderated.*

We now include two new benchmarks, one where we embedded plasmids into an e coli genome to better simulate actual discovery practices, and one where we tested whether or not conserved elements show up as false positives. See details in response to Review 1.

• *Fig 3. There seem to be light blue or gray bands that are not explained in the key. Also, because of the way the figure is drawn, it is difficult to discern where the different methods agreed.*

We have redrawn the figure with a new key to make it more clear where the methods agreed. We furthermore provide a new Supp Fig 1 to explicitly highlight only areas of agreement.

• *"From these discordant results, we conclude that none of the methods are fully capturing the wide breadth of mobile genetic elements in nature." - This is difficult to assess in the absence of a truth set.*

We hope that our new false positive test convinces readers that all three methods are indeed finding MGEs, so we stand by our conclusion.

• *Have you considered using pangenome gene sets as the reference set rather than representative genomes? Seems like that would increase your sensitivity. Please discuss.*

See response to review 1. We now include a new discussion snippet about pangenome datasets.

Review 1

Review comments

This is a very interesting approach to finding mobile elements in genomes using near-exact match searches on subsequences of a genome, and evaluating the evolutionary distance between the organisms using the TimeTree of Life estimates.

Authors: We thank the reviewer for the positive assessment of the usefulness of the approach. We believe this methodology offers significant insights into elucidating the evolutionary dynamic of mobile elements, and can have great potential for bulk metagenomics analyses.

A near-exact match to a highly divergent organism suggests lateral gene transfer of that region. This is very promising work that also demonstrates that we still have work to do in terms of informatically predicting mobile elements in genomic data. The low amount of overlap in results between the three methods tested was surprising and intriguing. The one significant drawback

of this study is the lack of a robust truth set to test the three tools against. Could such a dataset be constructed? It likely would be a challenge to construct, but without one, conclusions about sensitivity or accuracy must be moderated.

We very much agree with the Reviewer that a robust truth set is important, though as the Reviewer states, it is difficult to construct one due to the diverse nature of mobile elements and the innate differences by which various classification software annotate genomes. We now include two new benchmarks to better assess both recall and false positive rate (FPR) across skandiver, geNomad, and MobileElementFinder:

- (Fig 5.): Our old isolated plasmid test for recall is now Fig 5b, where we just ran the plasmids through each of the three methods. This is of course unnatural for detecting new integrated MGEs within genomes, so in the new Fig 5a, we now artificially embed those plasmids into an *E. coli* genome with 10kbp separation between each plasmid, and:
- (Table 1.): We now take a set of known conserved elements from NCBI (accession IDs available on the provided Github repository) to test for false positives. For the five >10kbp conserved elements we extracted, none of them were false positives for any of the three methods. For all conserved elements including much smaller ones, when run as isolated sequence using the three methods, skandiver had a 2.2% FPR, geNomad had a 1.3% FPR, and MobileElementFinder had no false positives, though most of those sequences were quite small, and so not in the >10kbp regime we've restricted ourselves to in this manuscript. We originally also tried using random sequences as a false positive dataset, but that didn't seem realistic and resulted in a 0% FPR for all methods, which is why we switched to conserved elements in Table 1.

Fig 3. There seem to be light blue or gray bands that are not explained in the key. Also, because of the way the figure is drawn, it is difficult to discern where the different methods agreed.

Thank you for pointing this out. The light blue or gray bands were intended to represent areas of overlap between different software outputs, indicated by striped sections. To make this more clear, we have updated it by making the stripes thicker and more dispersed, to ensure they are distinct and no longer appear as solid blue or gray bands (as well as including a legend portion for these striped sections). Additionally, we have included Supp Figure 1 that provides detailed plots of the overlapping regions for the various genes depicted in this figure. We believe these changes will make the figure more comprehensible and clearly indicate where the different methods converge and diverge.

"From these discordant results, we conclude that none of the methods are fully capturing the wide breadth of mobile genetic elements in nature." - This is difficult to assess in the absence of a truth set.

We believe that the new truth set of the embedded known plasmids in Fig 5a now better supports this statement.

Have you considered using pangenome gene sets as the reference set rather than representative genomes?

Making use of the pangenome datasets is an excellent idea. We haven't thought specifically about using gene sets, but we are currently playing around with using a broader set of genomes than just GTDB, including more strain-specific genomes. One of the difficulties is that the TimeTree of Life may not include evolutionary divergence times between those genomes, so we've been exploring whether we can just use ANI as a rough proxy, but there are some additional technical challenges we've run into on that front. Definitely something we have been exploring in continuing work.

We now include a new discussion snippet about pangenome datasets (a modified and more formal version of the paragraph above).

Seems like that would increase your sensitivity. I also found the paper to be well written - easy to understand and following a logical progression.

We thank the Reviewer for the positive feedback on our writing.

Review 2

Review commentsThe preprint titled "skandiver: a divergence-based analysis tool for identifying intercellular mobile genetic elements" introduces a new bioinformatics tool, skandiver, designed to detect mobile genetic elements (MGEs) in genomic sequences without relying on gene annotation or curated databases. The tool utilizes genome fragmentation, average nucleotide identity (ANI), and divergence time to identify MGEs, offering a unique approach by focusing on evolutionary divergence rather than sequence similarity to known elements.

We thank the Reviewer for the positive assessment of our unique approach.
